# Gasdermin E promotes translocation of p65 and c-jun into nucleus in keratinocytes for progression of psoriatic skin inflammation

**DOI:** 10.1038/s41419-024-06545-5

**Published:** 2024-03-01

**Authors:** Fangyuan Long, Xuecui Wei, Yujie Chen, Min Li, Ni Lian, Shanshan Yu, Sihan Chen, Yong Yang, Min Li, Heng Gu, Xu Chen

**Affiliations:** 1 Jiangsu Key Laboratory of Molecular Biology for Skin Diseases and STIs, Hospital for Skin Diseases, Institute of Dermatology, Chinese Academy of Medical Sciences & Peking Union Medical College, 210042 Nanjing, Jiangsu China; 2 School of Public Health, Nanjing Medical University, 211166 Nanjing, Jiangsu China

**Keywords:** Psoriasis, Cell death

## Abstract

Gasdermin E (GSDME) has recently been identified as a critical executioner to mediate pyroptosis. While epidermal keratinocytes can initiate GSDME-mediated pyroptosis, the role of keratinocyte GSDME in psoriatic dermatitis remains poorly characterized. Through analysis of GEO datasets, we found elevated GSDME levels in psoriatic lesional skin. Additionally, GSDME levels correlated with both psoriasis severity and response to biologics treatments. Single-cell RNA sequencing (scRNA-seq) from a GEO dataset revealed GSDME upregulation in keratinocytes of psoriasis patients. In the imiquimod (IMQ)-induced psoriasis-like dermatitis mouse model, both full-length and cleaved forms of caspase-3 and GSDME were elevated in the epidermis. Abnormal proliferation and differentiation of keratinocytes and dermatitis were attenuated in *Gsdme*^-/-^ mice and keratinocyte-specific *Gsdme* conditional knockout mice after IMQ stimulation. Exposure of keratinocytes to mixed cytokines (M5), mimicking psoriatic conditions, led to GSDME cleavage. Moreover, the interaction between GSDME-FL and p65 or c-jun was significantly increased after M5 stimulation. GSDME knockdown inhibited nuclear translocation of p65 and c-jun and decreased upregulation of psoriatic inflammatory mediators such as IL1β, CCL20, CXCL1, CXCL8, S100A8, and S100A9 in M5-challenged keratinocytes. In conclusion, GSDME in keratinocytes contributes to the pathogenesis and progression of psoriasis, potentially in a pyroptosis-independent manner by interacting and promoting translocation of p65 and c-jun. These findings suggest that keratinocyte GSDME could serve as a potential therapeutic target for psoriasis treatment.

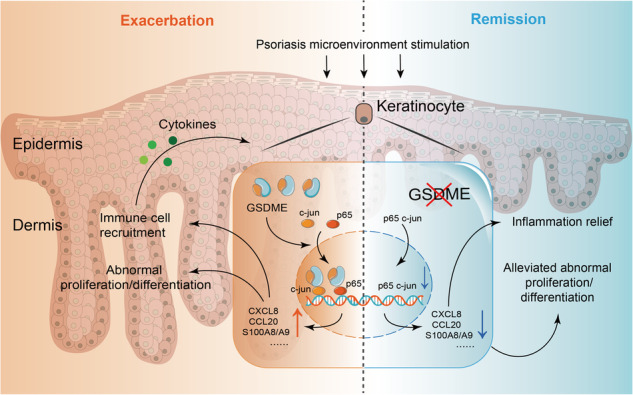

## Introduction

Psoriasis is a common, chronic immune-mediated skin disease affecting 2% of the population with red, scaly plaques [1]. Psoriasis substantially diminishes life quality and imposes significant psychosocial challenges on patients [2]. The pathogenesis of psoriasis is multifaceted. Recent research underscores the centrality of the IL-23/IL-17 axis, with biologics targeting IL-17, IL-23, and TNF-α revolutionizing the therapies [3, 4]. However, emerging findings spotlight that keratinocytes play a critical role in the initiation or amplification of inflammatory responses in psoriasis [5–7].

Pyroptosis, a form of regulated cell death, is defined as critically depending on the formation of plasma membrane pores mediated by the gasdermin protein family (GSDM), including GSDMA, GSDMB, GSMDC, GSDMD, and GSDME [8]. Our recent investigation revealed a correlation between GSDMD-mediated pyroptosis and hyperproliferation and aberrant differentiation of keratinocytes in psoriasis, and targeting GSDMD could alleviate the severity of psoriasis-form dermatitis [9]. Besides executing pyroptosis, GSDM has been implicated in exhibiting other biological effects. For example, He et al. [10] reported a new non-pyroptosis role of GSDMD. 13-kD N-terminal fragment of GSDMD can translocate to the nucleus and act as a regulatory hub for immune tolerance to food in small intestinal epithelial cells (IECs). Additionally, in IECs, Rana et al. [11] found that non-cleaved form GSDMB full-length (FL) relocates to the plasma membrane, influencing cell proliferation, migration, and cellular adhesion rather than inducing pyroptotic pores formation. In the realm of skin disorders, Huang et al. [12] reported that GSDMA FL mediates normal epidermal differentiation and cornification, and *Gsdma1/a3* deficiency could promote T helper 2 (Th2) inflammatory response in atopic dermatitis. Kusumaningrum et al. [13] discovered that ultraviolet radiation (UV) augments GSDMC FL expression in keratinocytes, which boosts UV-induced matrix metalloproteinases-1 (MMP-1) level. These findings indicate the potential multifaceted roles of GSDM in the biology and pathology of skin disorders, potentially involving both canonical pyroptosis-dependent and pyroptosis-independent mechanisms. Yet, whether other gasdermin proteins except GSDMD involve in the pathogenesis and progression of psoriasis remains unknown.

Keratinocytes, as the body’s primary defensive barrier, are more susceptible to various harmful external stimuli, leading to cell damage or even death. In the process of regulated cell death, the damaged cell often releases pro-inflammatory mediators to regulate immune and inflammation responses [14]. Our previous study suggested that necroptosis-mediated High mobility group box 1 (HMGB1) release from keratinocytes facilitates the progression of allergic contact dermatitis [15]. In another study, Vats et al. found that ferroptosis is the main death mechanism for releasing damage-associated molecular patterns (DAMPs) like HMGB1 in keratinocytes exposed to UVB radiation [16]. Furthermore, both necroptosis and ferroptosis in keratinocytes have been implicated in psoriasis and IMQ-induced psoriasis-like dermatitis, through the release of inflammatory cytokines such as S100A8, S100A9, HMGB1, IL-33, IL-1β, and IL-6 [17, 18]. However, the role of GSDME in keratinocytes within inflammatory skin diseases, whether through pyroptosis-mediated secretion of inflammatory factors or a non-pyroptosis mechanism, remains unclear.

In this study, by analyzing bioinformatic databases, we first compared the difference of GSDME in transcriptional level among normal skin of healthy people, lesional skin and non-lesional skin of psoriatic patients. Moreover, we also assessed GSDME alterations post-biologics therapy. Then we detected GSDME expression in imiquimod (IMQ)-induced psoriasis-like dermatitis mouse model. Furthermore, *Gsdme* knockout (KO) mice (*Gsdme*^*-/-*^ mice) and keratinocyte-specific *Gsdme* conditional knockout (cKO) mice were used to explore the role of GSDME in psoriasis-like dermatitis. GSDME knockdown keratinocytes were established to investigate the mechanism by which GSDME in keratinocytes participates in psoriasis-like inflammation. Furthermore, we verified the interaction between GSDME and key proinflammatory transcriptional factors.

## Results

### Pyroptosis key gene GSDME correlates with psoriasis

**Fig. 1:**
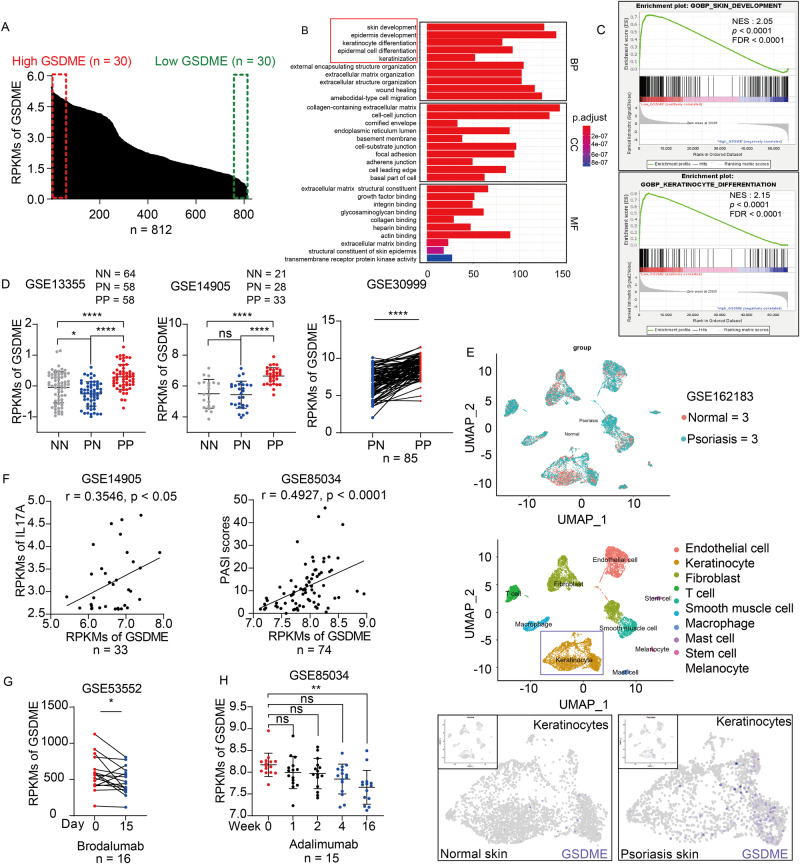
Pyroptosis key gene GSDME correlates with psoriasis. **A** 812 normal skin tissues from GTEx database were classified into “High GSDME” group (30 cases) and “Low GSDME” group (30 cases) based on Reads Per Kilobase per Million mapped reads (RPKMs) of GSDME. **B** Heat map from hierarchical cluster analysis revealed the differentially expressed genes (DEGs) between two groups were enriched in keratinocyte functions. **C** GSEA analysis enriched GO BP plots, including skin development and keratinocyte differentiation, which were positively correlated with the “Low GSDME” group. **D** Comparison of GSDME mRNA RPKMs among normal skin from healthy controls (NN), non-lesional skin from patients (PN) and lesional skin from patients (PP) using datasets GSE13355, GSE14905, and GSE30999. **E** In keratinocytes, GSDME expression was higher in lesions of psoriasis patients compared to that of healthy people, from scRNA-seq data of GSE162183. **F** Correlation between GSDME level and psoriatic indicators (IL-17A mRNA level) or PASI scores in psoriasis patients were analyzed by Pearson or Spearman correlation analysis from GSE14905 and GSE85034. **G**, **H** The changes of GSDME mRNA level in lesional skin tissues of psoriasis patients (from GSE53552 and GSE85034) after treatment with Brodalumab or Adalimumab. BP biological process, CC cellular component, MF molecular function, NES Normalized Enrichment Score, FDR False Discovery Rate. ns not significant. *p < 0.05, **p < 0.01, ***p < 0.001, ****p < 0.0001.

To investigate GSDME’s role in the skin, we analyzed the RNA-seq data from 812 normal skin tissue samples sourced from the Genotype-Tissue Expression (GTEx) Portal database [19]. We categorized the RNA profiles based on GSDME mRNA levels, selecting 30 samples with notably high expression and 30 samples with notably low expression. These were subsequently grouped into “High GSDME” and “Low GSDME” groups (Fig. 1A). Then we compared the two groups’ gene expression profiles in the gene ontology (GO) and found that the differentially expressed genes (DEGs) of the two groups have a close association with skin development, epidermis development, keratinocyte differentiation, epidermal cell differentiation, and keratinization (Fig. 1B). Further analysis through Gene set enrichment analysis (GSEA) confirmed the expression level of GSDME may play an important role in the regulation of skin development and keratinocyte differentiation. And they are upregulated in the “Low GSDME” group. (Fig. 1C).

Previous studies have shown the relevance of GSDME-mediated epithelial cell pyroptosis to inflammatory diseases [20, 21]. Tan et al. reported that GSDME-mediated intestinal epithelial-cell pyroptosis participated in the pathogenesis of Crohn’s disease by releasing proinflammatory cytokines [20]. Li et al. [21] found that GSDME-mediated pyroptosis in renal tubular cells led to renal tubular injury, causing subsequent hydronephrosis, inflammation, and fibrosis. However, the role of GSDME in keratinocytes for pathogenesis of psoriasis remains unclear. To investigate the correlation between GSDME and psoriatic dermatitis, we first performed bioinformatics analysis. We analyzed GEO datasets GSE13355 from Nair’s study [22], GSE14905 from Yao’s study [23], and GSE30999 from Suárez-Fariñas’s study [24], we observed elevated GSDME mRNA levels in skin biopsy samples from lesional skin of moderate to severe psoriasis (PP) compared to both normal skin from healthy controls (NN) and non-lesional skin of the patients (PN) [22, 23], or just non-lesional skin of the patients (PN) [24] (Fig. 1D). By analyzing of scRNA-seq dataset GSE162183 from Gao Y’s study [25], GSDME levels were elevated in keratinocytes from psoriasis patients compared to healthy controls (Fig. 1E). Furthermore, from analysis of GSE14905 from Yao’s study [23] and GSE85034 from Correa’s study [26], we observed a positive correlation between GSDME mRNA levels and IL-17A mRNA levels in skin tissue. Additionally, GSDME mRNA levels were positively correlated with psoriatic severity as assessed by the Psoriasis Area and Severity Index (PASI) score (Fig. 1F). Interestingly, post-treatment reductions in GSDME mRNA levels were noted in psoriatic skin following both anti-IL17 and anti-TNF-α treatment. For instance, GSDME mRNA levels in skin biopsies of psoriasis patients declined on Day 15 after Brodalumab biological agents, through analyzing GSE53552 from Russell’s study [27] (Fig. 1G). In addition, by analyzing GSE85034 from Correa’s study [26], we found GSDME mRNA levels were downregulated after Adalimumab anti-TNF-α therapy on Day 16 (Fig. 1H). Taken together, these data suggested that pyroptosis key gene GSDME might play a crucial role in the pathogenesis and progression of psoriasis.

### Protein level and cleavage of GSDME are elevated in IMQ-induced psoriasis-like dermatitis

**Fig. 2:**
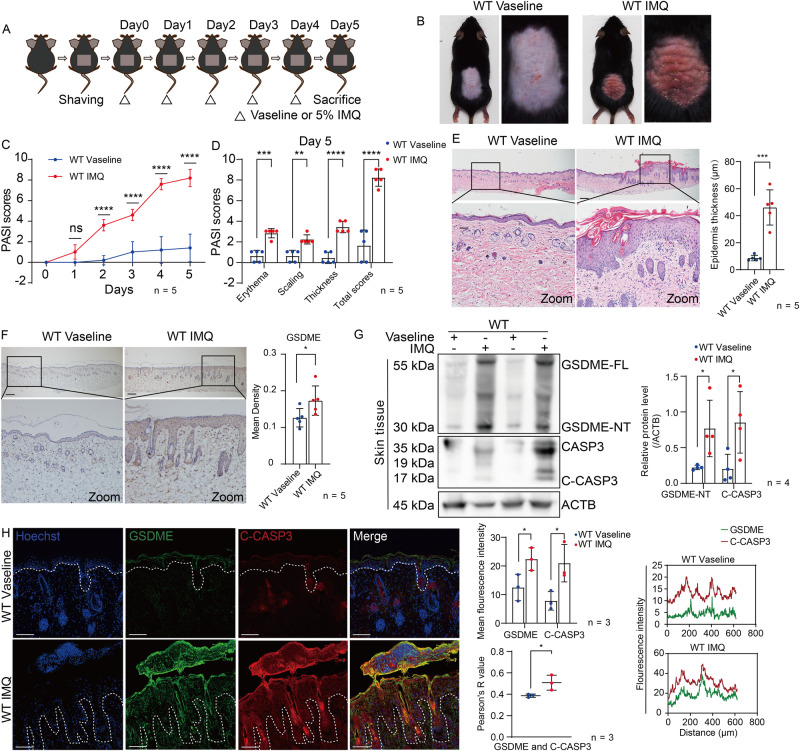
Protein level and cleavage of GSDME are elevated in IMQ-induced psoriasis-like dermatitis. **A** Protocol for inducing psoriasis-like dermatitis in mice using IMQ. **B** Representative images of dorsal skin from WT mice treated with either Vaseline or IMQ. **C** Evaluation of PASI scores for two groups from day 0 to day 5. **D** Day 5 scores for erythema, scaling, thickness, as well as total PASI scores were shown. **E** Representative H&E-stained images of skin sections. Statistical analysis of epidermal thickness was shown. **F** GSDME FL protein was presented by immunohistochemistry assay. Statistical analysis on mean staining intensity was shown. **G** Proteins of GSDME-FL, GSDME-NT, CASP3, and C-CASP3 were detected by western blotting assay. Statistical analysis of interested protein levels was shown. ACTB was used as a loading control. **H** GSDME FL and C-CASP3 were presented by immunofluorescence assay. Statistical analysis included mean immunofluorescence intensity, colocalization, and Pearson correlation analysis between GSDME FL and C-CASP3. Scale bar is 200 μm in **E**, **F**. Scale bar is 100 μm in **H**. WT wide type, GSDME-FL GSDME full length, GSDME-NT GSDME N-terminal, CAPS3 caspase-3, C-CAPS3 cleaved caspase-3. ns not significant. *p < 0.05, **p < 0.01, ***p < 0.001, ****p < 0.0001.

To further explore whether GSDME is activated in psoriasis, we applied imiquimod (IMQ) cream on the wide type (WT) C57BL/6 mice dorsal skin for 5 consecutive days to establish a psoriasis-like dermatitis mouse model [28] (Fig. 2A). Significant erythema, scaling, and thickness were observed in IMQ-treated mice (Fig. 2B). These observations were quantified and presented as the accumulated PASI score (Fig. 2C, D). Histopathological examination revealed typical features of psoriasis-like dermatitis, including abnormally increased proliferation, epidermal acanthosis, and parakeratosis (Fig. 2E). Consistent with prior GEO dataset analyses from psoriasis skin samples [22–24], we observed elevated GSDME protein levels in the epidermis of IMQ-treated mice through immunohistochemistry study (Fig. 2F). Through Western blotting assay, we further confirmed elevated levels of GSDME full-length (GSDME-FL) and GSDME N-terminal (GSDME-NT) in IMQ-treated mice compared to those treated with Vaseline (Fig. 2G). GSDME-NT, a pyroptosis-inducing fragment, indicated the activation of GSDME. As caspase-3 is a known upstream regulator leading to GSDME cleavage, activation of caspase-3 was also evident in IMQ-treated mice (Fig. 2G). Moreover, colocalization of GSDME and cleaved-caspase-3 in the epidermis of IMQ-treated mice was observed through immunofluorescence study (Fig. 2H). Collectively, these results indicated that caspase-3-mediated GSDME activation occurs in the epidermis of IMQ-induced psoriasis-like dermatitis.

### GSDME deficiency alleviates IMQ-induced psoriasis-like dermatitis in mice

**Fig. 3:**
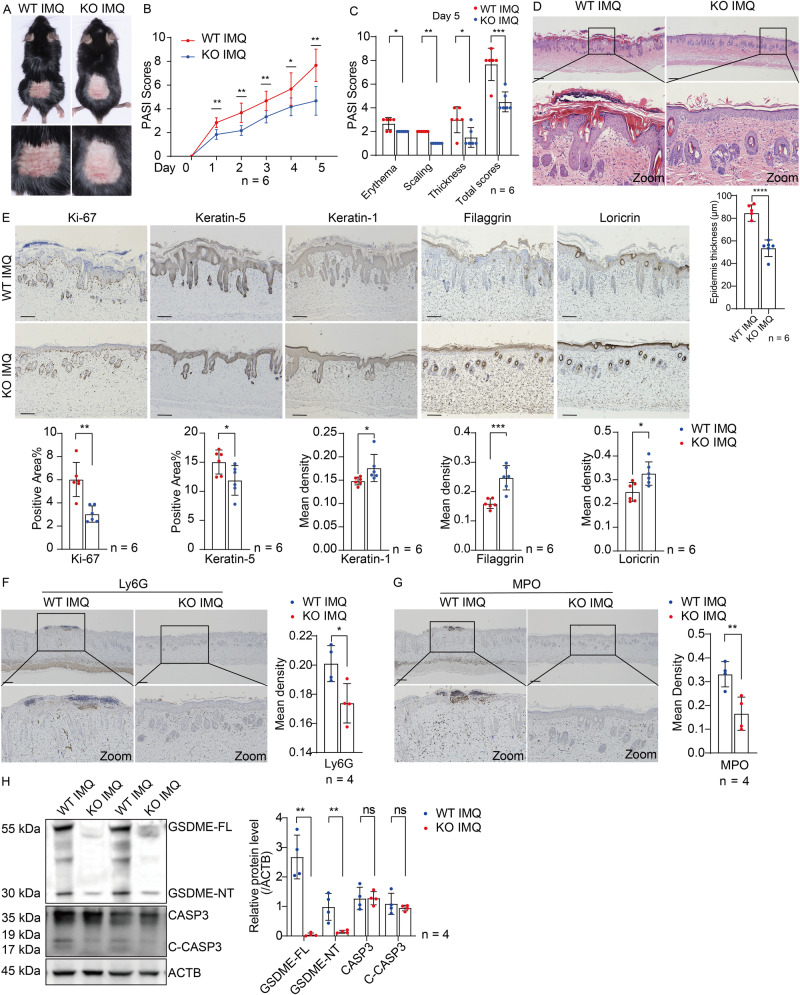
GSDME deficiency alleviates IMQ-induced psoriasis-like dermatitis in mice. **A** WT and *Gsdme*^−/−^ mice were treated with IMQ cream. Representative images of dorsal skin lesion were presented. **B** PASI scores progression of above two groups from day 0 to day 5. **C** Scores for erythema, scaling, and thickness, as well as PASI scores on day 5. **D** Representative H&E-stained images of skin sections. Statistical analysis of epidermal thickness was shown. **E** Immunohistochemistry study showed markers of keratinocyte proliferation (Ki-67 and Keratin-5) and differentiation (Keratin-1, Filaggrin, and Loricrin). Statistical analysis of positive area percentage or mean staining intensity of interested proteins was shown. **F**, **G** Neutrophil infiltration was evaluated by detecting Ly6G and MPO through immunohistochemistry study. Statistical analysis of mean staining intensity was shown. **H** Proteins of GSDME-FL, GSDME-NT, CASP3, and C-CASP3 were detected by western blotting assay. Statistical analysis of interested protein levels was shown. ACTB served as a loading control. Scale bar is 200 μm in **D**, **F**, and **G**. Scale bar is 100 μm in **E**. WT wide type, GSDME-FL GSDME full length, GSDME-NT GSDME N-terminal, CAPS3 caspase-3, C-CAPS3 cleaved caspase-3. ns not significant. *p < 0.05, **p < 0.01, ***p < 0.001, ****p < 0.0001.

To further elucidate the role of GSDME in the pathogenesis of psoriasis, *Gsdme*^*-/-*^ mice were used to determine whether GSDME deficiency could relieve the responses to IMQ stimulation. While we observed mild responses including erythema, scaling, and thickness in *Gsdme*^*-/-*^ mice after IMQ stimulation, these responses were significantly lower than those in the IMQ-treated WT mice from day 1 to day 5 (Fig. 3A–C). Moreover, histological analyses revealed a reduction in epithelial hyperproliferation and diminished inflammatory cell infiltration in the IMQ-treated *Gsdme*^*-/-*^ mice (Fig. 3D).

Next, we assessed the variations in a series of keratinocyte proliferation and differentiation markers between IMQ-treated WT mice and IMQ-treated *Gsdme*^*-/-*^ mice [29, 30]. However, compared to IMQ-treated WT mice, IMQ-treated *Gsdme*^*-/-*^ mice presented decreased Ki-67 expression, a reduced area of Keratin-5 expression in the epidermis, and a restored expression of Keration-1, Filaggrin, and Loricrin in the upper layers of epidermis (Fig. 3E). In addition, the infiltration of neutrophils and the formation of Munro microabscess, which were indicated by the detection of Ly6G and myeloperoxidase (MPO), were alleviated in IMQ-treated *Gsdme*^*-/-*^ mice compared with IMQ-treated WT mice (Fig. 3F, G).

Through the detection of GSDME in the lysate of skin tissue by western blotting, we confirmed that GSDME protein was not expressed in *Gsdme*^*-/-*^ mice. However, IMQ-induced cleavage of caspase-3 showed no significant difference between *Gsdme*^*-/-*^ mice and WT mice after IMQ stimulation (Fig. 3H).

Taken together, these results suggested that GSDME deficiency suppresses IMQ-induced psoriasis-like dermatitis.

### GSDME of keratinocyte is involved in IMQ-induced psoriasis-like dermatitis

**Fig. 4:**
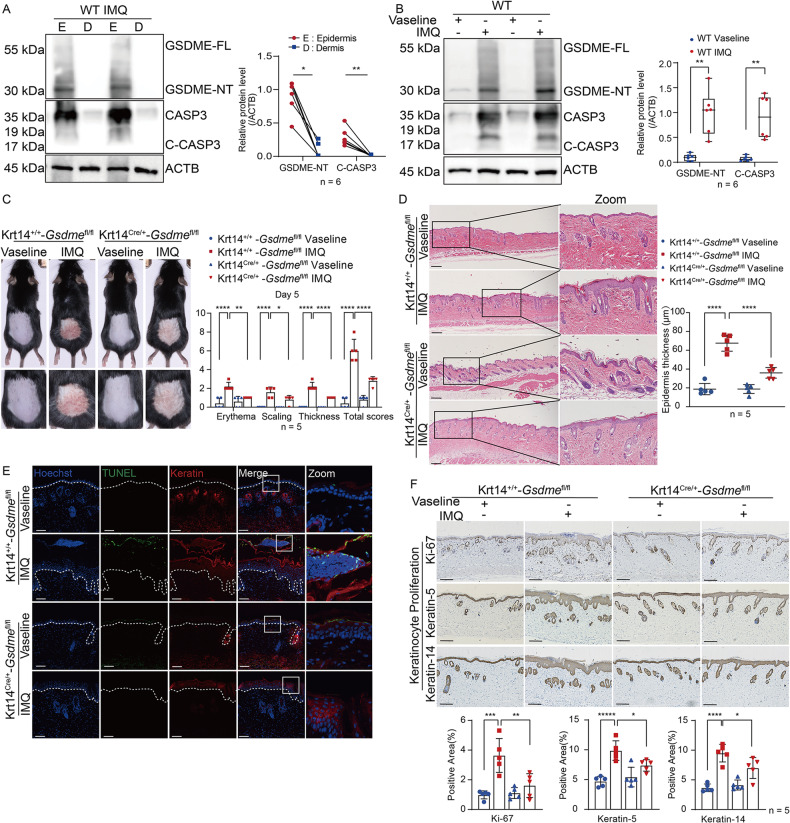

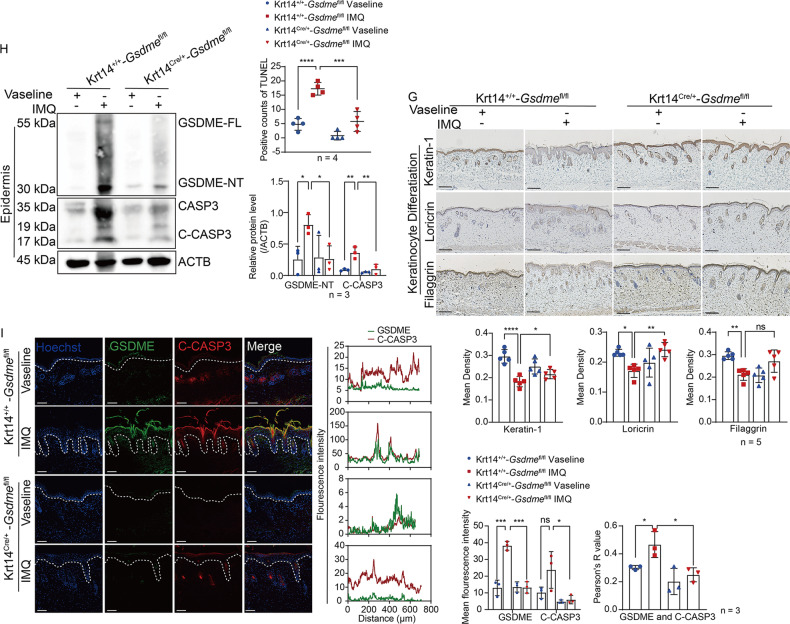
GSDME of keratinocyte is involved in IMQ-induced psoriasis-like dermatitis. **A** Proteins of GSDME-FL, GSDME-NT, CASP3, and C-CASP3 were detected in epidermis and dermis lysate from IMQ-treated WT mice through western blotting assay, respectively. Statistical analysis of interested protein levels was shown. **B** The above proteins were detected in epidermis lysate from WT mice after either Vaseline or IMQ treatment by western blotting assay. Statistical results of interested protein levels were shown. **C** Krt14^Cre/+^-*Gsdme*^fl/fl^ mice and their control mice received either Vaseline or IMQ treatment. Day 5 PASI scores were displayed. **D** Representative H&E-stained images of skin sections showed histological features. Statistical analysis of epidermal thickness was shown. **E** TUNEL assay was used to detect dead cells. Statistical analysis of dead cell counts was shown. **F**, **G** Immunohistochemistry study showed markers of keratinocyte proliferation (Ki-67, Keration-5, and Keration-14) and differentiation (Keration-1, Filaggrin, and Loricrin). Statistical analysis of positive area percentage or mean staining intensity was shown. **H** Proteins of GSDME-FL, GSDME-NT, CASP3, and C-CASP3 in epidermis lysate of cKO mice were detected by western blotting assay. Statistical analysis of interested protein levels was shown. **I** GSDME FL and C-CASP3 were detected by immunofluorescence assay. Statistical analysis of mean immunofluorescence intensity, colocalization, and Pearson correlation analysis between GSDME FL and C-CASP3 were shown. Scale bar is 200 μm in **D**. Scale bar is 100 μm in **E**–**G** and **I**. WT wide type, GSDME-FL GSDME full length, GSDME-NT GSDME N-terminal, CAPS3 caspase-3, C-CAPS3 cleaved caspase-3. ns not significant, *p < 0.05, **p < 0.01, ***p < 0.001, ****p < 0.0001.

To ascertain the role of GSDME more precisely in keratinocytes of psoriatic dermatitis, we crossed *Gsdme*-floxed mice with Krt14-Cre transgenic mice for selective ablation of *Gsdme* in keratinocytes to obtain conditional knockout mice (Krt14^Cre/+^-*Gsdme*^fl/fl^ mice) and its littermate controls (Krt14^+/+^-*Gsdme*^fl/fl^ mice). By western blotting assay, we first detected the distribution of GSMDE in the epidermis and dermis of skin tissue in IMQ-treated WT mice by extracting protein samples after separating skin samples. We found that both GSDME and caspase-3 were significantly higher in epidermis than dermis (Fig. 4A). Furthermore, we validated that the cleavage of GSMDE and caspase-3 was evident in the epidermis of WT mice after IMQ treatment (Fig. 4B).

Importantly, PASI scores of IMQ-treated Krt14 ^Cre/+^-*Gsdme*^fl/fl^ mice were found to be lower compared to those of IMQ-treated Krt14^+/+^-*Gsdme*^fl/fl^ mice (Fig. 4C). Moreover, a reduction in epidermal hyperproliferation and inflammatory cell infiltration was observed in IMQ-treated Krt14^Cre/+^-*Gsdme*^fl/fl^ mice (Fig. 4D). By terminal deoxynucleotidyl transferase dUTP nick end labeling (TUNEL) test for evaluating cell death, we observed that the level of dead keratinocytes in IMQ-treated Krt14^Cre/+^-*Gsdme*^fl/fl^ mice was significantly lower than that in IMQ-treated Krt14^+/+^-*Gsdme*^fl/fl^ mice (Fig. 4E), suggesting inhibition of GSDME-mediated keratinocytes cell death. We also observed that increased expression of proliferative indicators Ki-67, Keratin-5, and Keratin-14 in epidermis were inhibited in IMQ-treated Krt14^Cre/+^-*Gsdme*^fl/fl^ mice by immunohistochemistry studies (Fig. 4F). As well as, abnormal expression of keratinocyte differentiation markers Keration-1, Filaggrin, and Loricrin were relatively recovered (Fig. 4G). Through western blotting assay, we assessed that the level of GSDME-NT was descended in epidermis lysate of IMQ-treated Krt14^Cre/+^-*Gsdme*^fl/fl^ mice, compared with IMQ-treated Krt14^+/+^-*Gsdme*^fl/fl^ mice (Fig. 4H). By immunofluorescence study, we verified specific deficiency of GSDME in the epidermis of Krt14^Cre/+^-*Gsdme*^fl/fl^ mice (Fig. 4I). In addition, the colocalization of GSDME and cleaved caspase-3 appeared to be diminished in the epidermis of IMQ-treated Krt14^Cre/+^-*Gsdme*^fl/fl^ mice (Fig. 4I).

Collectively, these findings revealed that GSDME of keratinocytes is involved in the pathogenesis of IMQ-induced psoriasis-like dermatitis.

### GSDME in keratinocytes promotes psoriasis-like skin inflammation

**Fig. 5:**
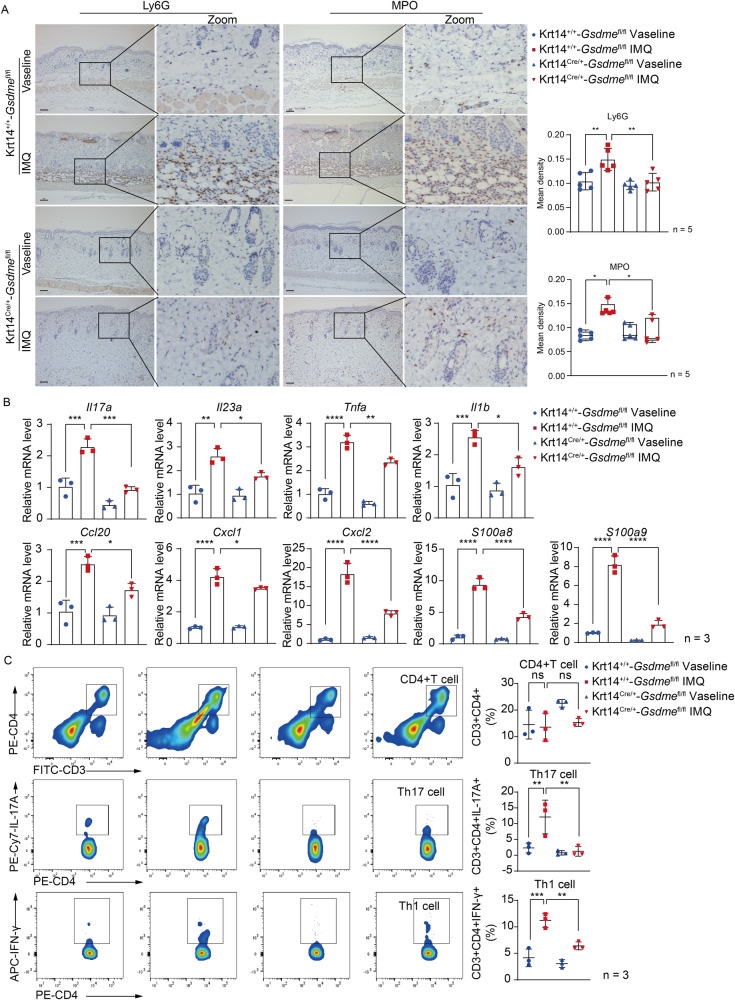
GSDME in keratinocytes promotes psoriasis-like skin inflammation. **A** Neutrophil infiltration was evaluated by detecting Ly6G and MPO through immunohistochemistry study. Statistical analysis of mean staining intensity was shown. **B** Quantitative real-time PCR analysis was performed to determine the mRNA levels of *Il17a, Il23a, Tnfa, Il1b, Ccl20, Cxcl1, Cxcl2, S100a8, and S100a9*. Statistical analysis was shown. **C** The percentage of CD4+T cells (CD3+CD4+), Th1 cells (CD3+CD4+IFN-γ+), and Th17 cells (CD3+CD4+IL-17A+) in spleen was determined by flow cytometry. Statistical analysis of specific cell percentages was shown. Scale bar represents 100 μm in **A**. ns not significant, *p < 0.05, **p < 0.01, ***p < 0.001, ****p < 0.0001.

To explore the function of keratinocyte GSDME in responses to psoriasis-like immune microenvironment, we first detected infiltration and activation of neutrophils by staining Ly6G and MPO in situ. We found that increases in infiltration and activation of neutrophils in IMQ-treated Krt14^+/+^-*Gsdme*^fl/fl^ mice were inhibited in IMQ-treated Krt14^Cre/+^-*Gsdme*^fl/fl^ mice (Fig. 5A). Through determining mRNA levels of some inflammatory cytokines, chemokines, and damage-associated molecular patterns (DAMPs) in skin tissue by qRT-PCR, we found that increases of *Il17a, Il23, Tnfa, Il1b, Cxcl1, Cxcl2, Ccl20, S100a8*, and *S100a9* in IMQ-treated Krt14^+/+^-*Gsdme*^fl/fl^ mice were reduced in IMQ-treated Krt14^Cre/+^-*Gsdme*^fl/fl^ mice. These reductions implied that specific deficiency of GSDME in keratinocytes can alleviate psoriasis-like immune microenvironment (Fig. 5B). Importantly, we observed that the rise in counts of CD3+CD4+IL-17A+T cells (Th17 cells) and CD3+CD4+IFN-γ+T cells (Th1 cells) in spleen of IMQ-treated Krt14^+/+^-*Gsdme*^fl/fl^ mice, and the immune responses of T cells to psoriasis-like immune microenvironment were suppressed in IMQ-treated Krt14^Cre/+^-*Gsdme*^fl/fl^ mice (Fig. 5C). Given the above, these findings validate that specific deficiency of GSDME in keratinocytes restricts immune responses to psoriasis-like inflammation microenvironment.

### GSDME promotes the translocation of p65 and c-jun into nucleus to upregulate psoriatic inflammation

**Fig. 6:**
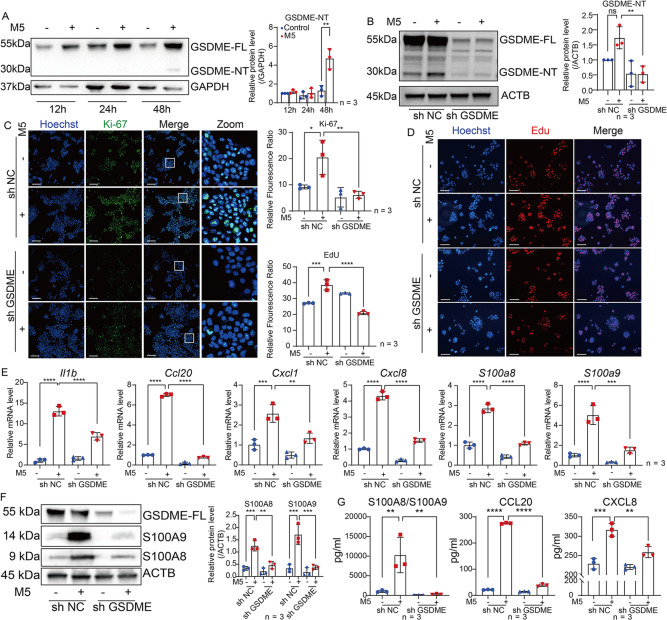

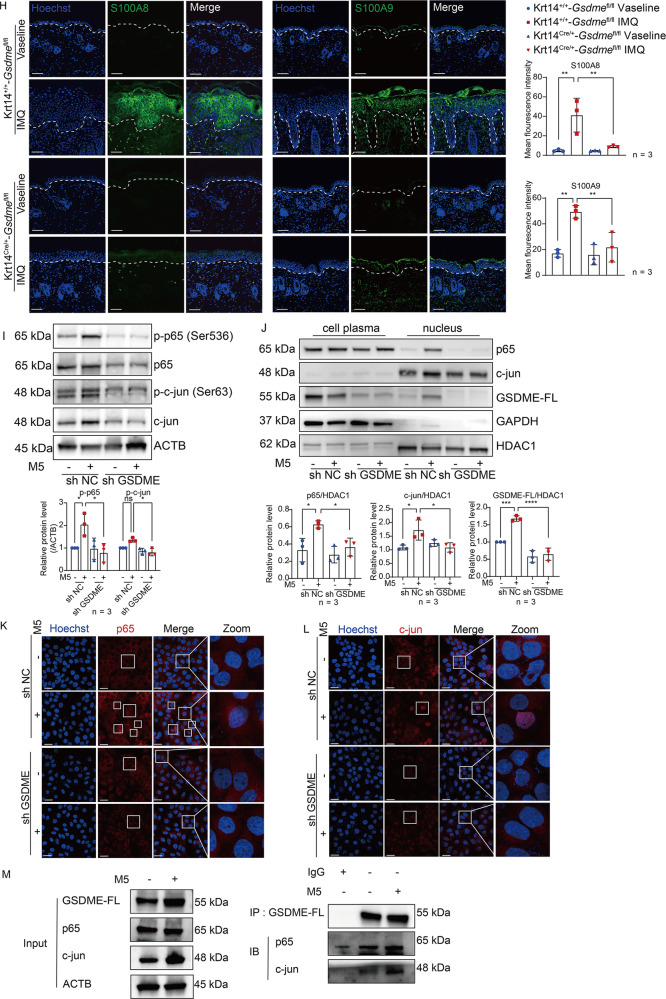
GSDME promotes translocation of p65 and c-jun into nucleus to upregulate psoriatic inflammation. **A** Proteins of GSDME FL and GSDME NT were detected by western blotting in HaCaT cells at 12, 24, and 48 h after treatment with or without M5. Statistical analysis of interested protein levels was shown. **B** HaCaT cells were transfected with lentivirus vector containing either NC shRNA or GSDME shRNA, establishing GSDME knockdown cells (sh GSDME) and control cells (sh NC). Knockdown efficiency was evaluated using western blotting assay. Statistical analysis of GSDME was shown. **C**, **D** Immunofluorescence assay was used to detect Ki-67 expression or Edu-positive cells. Statistical analysis of mean immunofluorescence intensity was shown. **E** Quantitative real-time PCR analysis was performed to determine mRNA levels of *Il1b, Ccl20, Cxcl1, Cxcl8, S100a8*, and *S100a9*. Statistical analysis was shown. **F** Proteins of GSDME FL, S100A8, and S100A9 in cell lysate were detected by western blotting assay. Statistical analysis of interested protein levels was shown. **G** Secretion levels of S100A8/A9, CCL20, and CXCL8 were determined by ELISA assay. Statistical analysis was shown. **H** S100A8 and S100A9 were detected by immunofluorescence assay in mice skin sections, respectively. Statistical analysis was shown. **I** Proteins of p-p65, p65, p-c-jun, and c-jun in cell lysate were detected by western blotting assay. Statistical analysis of interested protein levels was shown. **J** Proteins of p65, c-jun, and GSDME-FL were detected by western blotting assay in lysate of cell plasma or nucleus, respectively. Statistical analysis of interested protein levels was shown. GAPDH and HDAC1 served as a loading control of cell plasma protein and nucleus proteins, respectively. **K**, **L** Immunofluorescence assay was used to detect translocation of p65 and c-jun into nucleus. **M** Co-immunoprecipitation assay was used to detect the interaction between GSDME-FL and p65 or c-jun. Scale bar represents 100 μm in **C**, **D**, **H**, **K** and **L**. ns not significant, *p < 0.05, **p < 0.01, ***p < 0.001, ****p < 0.0001.

To further investigate whether GSDME cleavage in keratinocytes can be induced when keratinocytes are exposed to stimulation of psoriasis-like immune environment, HaCaT cells, an immortal human keratinocyte line, were exposed to a mixture of TNF-α, IL-17A, IL-22, Oncostatin M (OSM), IL1-α (Mixture 5, M5). This treatment serves as an in vitro model of skin inflammation replicating psoriatic features [31, 32]. 48 h after M5 treatment, we detected an increase of GSDME-NT in lysate of HaCaT cells by western blotting assay (Fig. 6A). Then we transfected HaCaT cells with lentivirus vectors containing GSDME short hairpin RNA (shRNA) to produce GSDME knockdown cells (sh GSDME) or with vectors containing a nonsense control (NC) shRNA as a control (sh NC). We verified that GSDME-FL was decreased in sh GSDME HaCaT cells. And GSDME-NT cannot be notably increased in M5-treated sh GSDME HaCaT cells compared to M5-treated sh NC (Fig. 6B).

Previous studies showed that M5 stimulation can induce proliferation of keratinocytes cultured in vitro [9, 33]. To ascertain whether GSDME plays an important role in keratinocyte responses to psoriasis-like stimulation, we first detected proliferative makers including Ki-67 and 5-Ethynyl-2’-deoxyuridine (EdU). Our findings showed that the positive cell counts of Ki-67 and EdU in sh GSDME HaCaT cells were lower than those in sh NC HaCaT cells after M5 treatment (Fig. 6C, D). Next, we discovered that M5-induced transcriptional upregulation of certain cytokines (IL-1β), chemokines (CCL20, CXCL1, and CXCL8), and DAMPs (S100A8 and S100A9) were restrained in sh GSDME HaCaT cells (Fig. 6E). Collectively, these results showed that GSDME might facilitate responses of keratinocyte to psoriasis-like stimulation.

Recently, S100A8 and S100A9 were proven to contribute to psoriatic skin inflammation and could be a biomarker of psoriasis severity [34, 35]. We analyzed ScRNA-seq dataset GSE162183 from Gao’s study [25] and found that both S100A8 and S100A9 were among the top 10 molecules expressed in lesional skin tissue of psoriasis patients, with notably high expression in keratinocytes (Supplemental Fig. 1). Therefore, we explored whether S100A8 and S100A9 participate in the function of GSDME in keratinocytes for promoting psoriasis-like dermatitis. We found that in accordance with the results of transcriptional study, M5-induced increases of S100A8 and S100A9 proteins at cellular level were significantly suppressed in sh GSDME HaCaT cells compared to sh NC HaCaT cells (Fig. 6F). Moreover, using Enzyme-linked immunosorbent assay (ELISA), we found that GSDME knockdown also significantly inhibited M5-induce secretion of S100A8/A9 (Fig. 6G). Importantly, by immunofluorescence study, we observed that S100A8 and S100A9 were significantly increased in epidermis of IMQ-treated Krt14^+/+^-*Gsdme*^fl/fl^ mice, however, the effects were alleviated in Krt14^Cre/+^-*Gsdme*^fl/fl^ mice (Fig. 6H).

NF-κB and MAPK pathways in keratinocytes are known to induce the expression of numerous proinflammatory factors including S100A8 and S100A9, as well as chemokines and cytokines such as CXCL1, CXCL2, CXCL5, CCL20, IL-6, and G-CSF [36–38]. Interestingly, through an RNA-seq study of total RNA samples from the epidermis of IMQ-treated Krt14^+/+^-*Gsdme*^fl/fl^ mice and IMQ-treated Krt14^Cre/+^-*Gsdme*^fl/fl^ mice, we found that significant difference in the activation of NF-κB and MAPK pathways between two groups (Supplemental Fig. 2). Thus, we further compared the difference of two pathways between sh GSDME HaCaT cells and sh NC HaCaT cells after M5 treatment, we found that M5-induced increases of phosphorylated p65 (at Ser 536 site) and c-jun (at Ser 63 site) in sh NC HaCaT cells were inhibited in sh GSDME HaCaT cells (Fig. 6I). Through both western blotting study (acquiring protein samples after separation of cell plasma and nucleus) and immunofluorescence assay, we found that M5-induced translocation of p65 and c-jun from cell plasma into nucleus was decreased in sh GSDME HaCaT cells compared to sh NC HaCaT cells (Fig. 6J–L). Moreover, we further confirmed that the interaction between GSDME-FL and p65 or c-jun was increased after M5 stimulation, as demonstrated by our co-immunoprecipitation assay results (Fig. 6M). These findings indicated that GSDME can promote the translocation into the nucleus of p65 and c-jun, and GSDME knockdown not only inhibits activation of p65 and c-jun but also reduces their translocation.

Taken together, GSDME might facilitate the nuclear translocation of key transcription factors, such as p65 and c-Jun, to regulate the synthesis and secretion of downstream inflammatory mediators.

## Discussion

This study reported that GSDME transcriptional levels were increased in psoriasis lesional skin and positively correlated with psoriasis progression. In IMQ-induced psoriasis-like dermatitis mice, full length, and cleavage of caspase-3 and GSDME were notably increased. In *Gsdme*^-/-^ mice and keratinocyte-specific *Gsdme* cKO mice, abnormal proliferation and differentiation of keratinocytes were recovered, and psoriasis-like dermatitis was ameliorated after IMQ stimulation. In M5-treated keratinocytes, GSDME FL in nucleus was increased. GSDME knockdown can decrease translocation into nucleus of p65 and c-jun and production and secretion of psoriatic inflammatory mediators, along with a decrease of GSDME FL in nucleus.

In this study, by analyzing bioinformation databases, we found that strong transcriptional level expression of GSDME is significantly more prominent in lesional skin of psoriasis than non-lesional skin of psoriasis patients and normal skin of healthy people. And GSDME levels were correlated with disease severity and treatment response. And we also observed elevated GSDME protein expression in psoriatic skin lesion from psoriasis-like dermatitis of mice. Thus, we speculate that GSDME expression might serve as a biomarker for psoriasis. Recently, Nowowiejska et al. discovered that GSDMD is more strongly expressed in skin and serum of psoriasis patients than healthy people [39]. Their study further indicated the potential association of gasdermin proteins with the pathogenesis and progression of psoriasis. Noteworthily, Tan et al. found that GSDME expression in small intestine tissue was positively associated with the clinical disease severity of Crohn’s disease [20]. However, the value of GSDME as one biomarker for assessing severity of psoriasis needs future clinical research to be validated.

Caspase-3 is recognized as a pivotal upstream activator of GSDME, cleaving GSDME specifically at Asp270 site [40]. Both extrinsic and intrinsic apoptosis signals lead to activation of caspase-3 to mediate apoptosis execution [41]. While caspase-3 activation of keratinocytes in psoriasis [42] and psoriasis-like dermatitis [17] has been reported, apoptosis in these keratinocytes remains suppressed. Interestingly, narrow-band UVB therapy was reported to alleviate lesions by activating caspase-3 and inducing apoptosis in psoriatic keratinocytes [43]. Although inhibition of GSDME and its cleavage can alleviate IMQ-induced skin lesion in *Gsdme*^*-/-*^ mice and keratinocyte-specific *Gsdme* cKO mice, caspase-3 activation was still observed in skin tissue. We speculate that the role of active caspase-3 in pathogenesis of psoriasis might primarily be to initiate GSDME cleavage and activation. In addition, TNF-α has been reported as the critical activator of caspase-3/GSDME-mediated pyroptosis [20, 21]. TNF-α is one of the most important cytokines in M5 used to mimic psoriatic inflammatory microenvironment in vitro. The actual role of TNF-α signaling in activation of caspase-3/GSDME in psoriatic keratinocytes needs more investigations to be clarified.

Recently studies reported many non-pyroptotic roles of gasdermin proteins. Non-pyroptotic cleavage fragment of GSDMD was reported to regulate gene expression. For example, He et al. found that the 13kD N-terminal GSDMD, a non-pyroptotic cleavage fragment of GSDMD, translocates to the nucleus to induce the transcription of class II major histocompatibility complex transactivator (CIITA) and major histocompatibility complex class II (MHCII) in IECs [10]. Additionally, Peng et al. demonstrated a new non-pyroptosis function of GSDMD FL. They found that hypoxia can induce GSDMD FL to translocate into nucleus of colorectal tumor cells to interact with poly (ADP-ribose) polymerase-1 (PARP-1), which blocks DNA repair function of PARP-1 to accelerate apoptosis [44]. Interestingly, in this study, we found that GSDME FL has a basic protein level in nucleus of keratinocytes (Fig. 6J). Furthermore, M5 treatment mimicking the psoriatic inflammatory environment can increase protein level of GSDME FL in the nucleus. Accompanying by a decline of GSDME FL protein in nucleus due to GSDME knockdown, we observed that M5-induced translocation of p65 and c-jun into nucleus was inhibited. More importantly, we observed a significant increase in the interaction between GSDME and p65 or c-jun after M5 stimulation (Fig. 6M). Taken together, aside from the reported role in pyroptosis, GSDME FL probably functions as a regulator to interact with crucial proinflammatory transcription factors such as p65 and c-jun and facilitate their translocation to the nucleus in psoriatic inflammation. However, the actual interaction mechanism needs further investigation.

Our study demonstrated that GSDME contributes to pathogenesis and progression of psoriatic skin inflammation, which might be mediated by a pyroptosis-independent manner. However, we did not clarify the actual interaction mechanism between GSDME and the key psoriasis-related transcription factor p65 and c-jun. Moreover, we did not elucidate the function of keratinocyte pyroptosis caused by GSDME cleavage in psoriatic inflammation responses. Given the emerging recognition of GSDME as a potential therapeutic target for inflammatory diseases and the absence of any reported molecules or compounds that specifically target GSDME for disease treatment, it becomes essential to develop therapies targeting GSDME. As such, it is important to uncover the intricate mechanisms linked to functions of GSDME in either pyroptosis process or pyroptosis-independent effects.

## Materials and methods

### GETx and GEO database analysis

Perl software, R Studio software, and Gene Set Enrichment Analysis (GSEA) were used to analyze the mRNA levels of 812 healthy skin tissues from GTEx database. Psoriasis-related data from Gene Expression Omnibus (GEO) database were reanalyzed by R Studio software, the accession numbers were shown in the corresponding figures.

### Reagents and antibodies

Primary antibodies against DFNA5/GSDME N-terminal (#ab215191), DFNA5/GSDME C-terminal (ab221843), Cytokeratin 1 (#ab185628), loricrin (#ab85679), Ki67 (#ab16667), Cytokeratin5 (#ab52635), Cytokeratin14 (#ab119695), Ly6g (#ab238132), Myeloperoxidase (#ab208670), and wide spectrum Cytokeratin (#ab9377) were provided by Abcam (Cambridge, MA, USA). Primary antibodies against Caspase-3 (#9662 S), Cleaved-Caspase3 (#9579 S), NF-κB p65 (#8242), Phospho-NF-κB p65 (#3033), c-Jun (#9165), Phospho-c-Jun (#2361), GAPDH (#5174), β-Actin (#8457), HDAC1 (#34589) were provided by Cell Signaling Technology (Danvers, MA, USA). Primary antibodies against S100A8 (#abs136076) and S100A9 (#abs137076) were provided by Absin (Shanghai, China). The primary antibody against DFNA5 (sc-393162) was provided by Santa Cruz Biotechnology (Dallas, Texas USA). FITC Hamster Anti-Mouse CD3e (#553061), Leukocyte Activation Cocktail (#550583), Fixation/Permeabilization Kit (#554714) were provided by BD Biosciences (Franklin Lake, NJ, USA). Primary antibodies against Filaggrin (#905804), and PE-Cy7 Rat Anti-Mouse IL-17A (#506522) were provided by BioLegend (San Diego, California, USA). APC Rat Anti-Mouse IFN-γ (#17-7311-82), PE Rat Anti-mouse CD4 (#12-0041-82), Fixable Viability Dye eFluor™ 780 (#65-0865-14), immunofluorescent secondary antibodies including Alexa Fluor™ 488 (#A21202) and Alexa Fluor™ 633 (#A21071) were provided by Thermo Fisher Scientific (Waltham, MA, USA). The secondary antibody conjugated with HRP (#88652) was from Cell Signaling Technology. Secondary antibody conjugated with HRP (#M21008) for co-immunoprecipitation assay was from Abmart (Shanghai, China).

### Animal study

All mice used in this study were purchased from GemPharmatech (Nanjing, Jiangsu, China) including C57BL/6JGpt mice (wild type, WT), B6/JGpt*Gsdme*^em8Cd566/Gpt^ (*Gsdme*^−/−^ mice), Krt14^Cre/+^-*Gsdme*^fl/fl^ (keratinocyte-specific *Gsdme* cKO mice) and their littermate controls (Krt14 + /+-*Gsdme*^fl/fl^). All mice in experiments were between 6-8 weeks of age and weighted 15-25 g. All mice in our experiment were male mice. All mice were randomly allocated to different groups. All mice were applied with imiquimod cream (Sichuan MingXin Pharmaceutical Co. China) on dorsal skin to establish a psoriasis-like dermatitis model. All mice were sacrificed after IMQ applied for 5 consecutive days, and their tissues were collected. No mice were excluded from the analysis. The severity of skin lesions in mice was evaluated by PASI scores, as detailed in our previous study [9]. Two investigators conducted a blind assessment of the severity of skin lesions in mice.

### Cell culture, psoriatic model in vitro and cell transfection

Human keratinocyte cell line, HaCaT cells, were obtained from China Center for Type Culture Collection (Wuhan, China). HaCaT cells were cultured in Dulbecco’s modified Eagle’s medium (DMEM) containing 10% fetal bovine serum (FBS) (both from Gibco, CA, USA) in 5% CO_2_ environment at 37 °C. HaCaT cells were treated with 10 ng/mL recombinant human (rh) IL-17A (R&D, Minneapolis, USA), 10 ng/mL rh OSM (R&D), 10 ng/mL rh TNF-α (R&D), 10 ng/mL rh IL22 (R&D), and 10 ng/mL rh IL1-α (R&D) in combination for 6, 12, 24, or 48 h. Transfection was performed according to previously described methods (45). For GSDME knockdown, HaCaT cells were transfected with pGLVH1/GFP + Puro lentivirus vector containing NC shRNA (shNC sequence: 5’-TTCTCCGAACGTGTCACGT-3’) or GSDME shRNA (shGSDME sequence: 5’-GCAGAAGTGTGTGATCTCTGA-3’) (GenePharma, Shanghai, China). Stable knockdown HaCaT cells were obtained and selected by puromycin incubation.

### Histological analysis, Immunohistochemistry assay

Hematoxylin and eosin staining, epidermal thickness measurement, and immunohistochemistry assay were performed as described previously [45]. The mean optical densities of GSDME, Keratin-1, Filaggrin, Loricrin, Ly6G, and MPO, as well as the positive area of Ki67, Keration-5, Keratin-14 in immunohistochemistry images were analyzed using Image J software.

### Immunofluorescence assay, TUNEL assay, and EdU assay

Immunofluorescence assays were conducted as described previously [45]. TUNEL assays were performed with a TUNEL kit (A112-01, Vazyme, Nanjing, China) according to the manufacturer’s protocols. EdU assays were performed with a BeyoClick^TM^ EdU-555 kit (C0075S, Beyotime, Shanghai, China). The positive staining cell counts of TUNEL, mean immunofluorescence intensity of skin sections, and relative immunofluorescence ratio of Ki-67 and EdU were analyzed by Image J software. Colocalization analysis was according to the protocol of O’Brien et al’s study by Image J software [46].

### Separation of epidermis and dermis

The separation process was performed as described previously [45]. Briefly, dorsal skin samples were collected and immersed into an isolation buffer, 2 mg/ml dispase II (Sigma-Aldrich, St. Louis, MO, USA), overnight at 4 °C. We separated the epidermis from the dermis gently the next day.

### Western blotting assay

Protein samples were prepared, and western blotting assays were performed as described previously [45]. Primary antibodies and secondary antibodies for the interested proteins used in western blotting assays were described in reagents and antibodies section. Image J software was used to calculate the density of interested protein bands. All replications of western blotting bands were shown in Supplemental Fig. 3.

### RNA extraction and quantitative real-time PCR

Total RNA extraction of tissue or cells, quantitative real-time PCR, and relative levels of interesting mRNA calculation were performed as previously described [45]. The mRNA levels of target genes were normalized to that of GAPDH. The primers used for the target genes were listed in Table 1.

**Table 1:** Primer sequences in this study.

Gene	Forward_sequence (5’→3’)	Reverse_sequence (5’→3’)
Mice		
*Il17a*	TACCTCAACCGTTCCACGTC	ATGTGGTGGTCCAGCTTTCC
*Tnfa*	CCCTCACACTCAGATCATCTTCT	GCTACGACGTGGGCTACAG
*Il1b*	GCAACTGTTCCTGAACTCAACT	ATCTTTTGGGGTCCGTCAACT
*Il23a*	AATAATGTGCCCCGTATCCAGT	GCTCCCCTTTGAAGATGTCAG
*Cxcl1*	CTGGGATTCACCTCAAGAACATC	CAGGGTCAAGGCAAGCCTC
*Cxcl2*	CAGACAGAAGTCATAGCCAC	TTCCAGGTCAGTTAGCCTTG
*Ccl20*	ACTGTTGCCTCTCGTACATACA	GAGGAGGTTCACAGCCCTTTT
*S100a8*	GACAATGCCGTCTGAACTGG	GCTACTCCTTGTGGCTGTCTT
*S100a9*	ACCACCATCATCGACACCTTC	AAAGGTTGCCAACTGTGCTTC

Human		
*Il1b*	AGCTACGAATCTCCGACCAC	CGTTATCCCATGTGTCGAAGAA
*Ccl20*	TGACTGCTGTCTTGGATACACAGA	TGATAGCATTGATGTCACAGCCT
*Cxcl1*	AGTTTTACAGTGTTTCTGGCTTA	GATTTTCCAGTAAAGGTAGCCC
*Cxcl8*	GTCCTTGTTCCACTGTGCCT	GCTTCCACATGTCCTCACAA
*S100a8*	ATGCCGTCTACAGGGATGA	CTGCCACGCCCATCTTTA
*S100a9*	TCATCAACACCTTCCACCAATAC	ATCTTTTCGCACCAGCTCTTT

### RNA sequencing

RNA sequencing (RNA-seq) was used to measure the mRNA expression levels in the epidermis isolated from IMQ-challenged *Gsdme* cKO mice and their littermates. Sequencing and data analysis were performed by Novogene Bioinformatics Technology Co. (Beijing, China).

### Flow cytometry

Spleen tissue of mice was collected to separate lymphocytes, including CD4 + T cells, Th1 cells, and Th17 cells. The lymphocyte single-cell suspension was obtained by the method of grinding. FVS780 was used to stain dead cells. After the ordinal processes of stimulation and block, lymphocytes were stained with CD3 and CD4 monoclonal antibodies, Fix & Perm, and IFN-γ and IL-17A. The percentage of Th1 (CD3+CD4+IFN-γ ) and Th17 (CD3+CD4+IL17A+) were calculated from CD3+CD4+ cells. Flow cytometry was then performed using BD ACSerse™ (BD, USA). Data were analyzed using FlowJo software. The gating strategy was shown in Supplemental Fig. 4.

### ELISA

ELISA was done according to the manufacturer’s instructions. ELISA kits of S100A8/A9 (KE00177), CCL20 (KE00149), and IL-8 (KE00006) were purchased from Proteintech (Wuhan, China).

### Protein separation of nucleus and cytoplasm

NE-PER™ nuclear and cytoplasmic extraction reagents (Thermo Fisher Scientific) was used to separate protein sample of nucleus and cytoplasm according to the manufacturer’s protocols.

### Co-immunoprecipitation assay

Immunoprecipitation Kit with Protein A+G Agarose Gel (P2197, Beyotime) was used to detect the interaction between proteins according to the manufacturer’s protocols. Briefly, cells were lysed in a lysis buffer containing a protease inhibitor cocktail in the Kit. The cell lysate was incubated with the specific capture antibody (GSDME (#ab221843, Abcam) or normal IgG) at 4 °C overnight. The Protein A+G Agarose Gel was washed two times with 1×TBS. The Protein A+G Agarose Gel was incubated with cell lysates at room temperature for 1 h. The immunoprecipitant was mixed with 2×SDS loading buffer. The sample was heated at 95 °C for 5 min, then the supernatant was collected for Western Blot assay.

### Statistical analysis

Statistical analyses were performed in SPSS and GraphPad Prism softwares. All data underwent normality test by Kolmogorov–Smirnov test or the Shapiro-Wilk test, with normally distributed data presented as mean ± standard deviation (SD), and non-normally distributed data represented by median and interquartile range. Statistical analysis methods were as follows: (1) Two groups: if the data was normally distributed and met homogeneity of variance, the Independent Samples t-test was chosen; otherwise, the Wilcoxon signed-rank test was applied. (2) Paired samples: if the data was normally distributed, the Paired Samples t-test was selected; otherwise, the Wilcoxon signed-rank test was applied. (3) Three or more groups: if the data was normally distributed and met homogeneity of variance, one-way ANOVA was conducted, followed by Tukey’s test for multiple comparisons; otherwise, the Kruskal-Wallis H test was used, followed by the Bonferroni correction for multiple comparisons. (4) Correlation analysis: if the data was normally distributed, Pearson correlation analysis was chosen; otherwise, Spearman correlation analysis was used, with the correlation coefficient denoted as “*r*”. “*n* =” represented the number of biological replicates used in our study. *p* < 0.05 was considered as statistical significance.

### Supplementary information

Supplemental Figure 1

Supplemental Figure 2

Supplemental Figure 3

Supplemental Figure 4

Supplemental Figure Legends

Original WB DATA

checklist

## Data Availability

All datasets utilized in this study are available from GTEx database (https://www.gtexportal.org/) and GEO database (http:// www.ncbi.nlm.nih.gov/geo/). GEO: GSE13355, GSE14905, GSE30999, GSE162183, GSE85034, and GSE53552. All data presented in this manuscript are available upon request from corresponding authors.

## Code availability

Code details are available upon reasonable request. All Code details used in this manuscript are available upon request from the corresponding authors.
