# Supplementary figures and images for: Gasdermin E promotes translocation of p65 and c-jun into nucleus in keratinocytes for progression of psoriatic skin inflammation
Source: Cell Death Dis. 2024 Mar 1;15(3):180. doi: 10.1038/s41419-024-06545-5 (PMC10907691; doi:10.1038/s41419-024-06545-5)

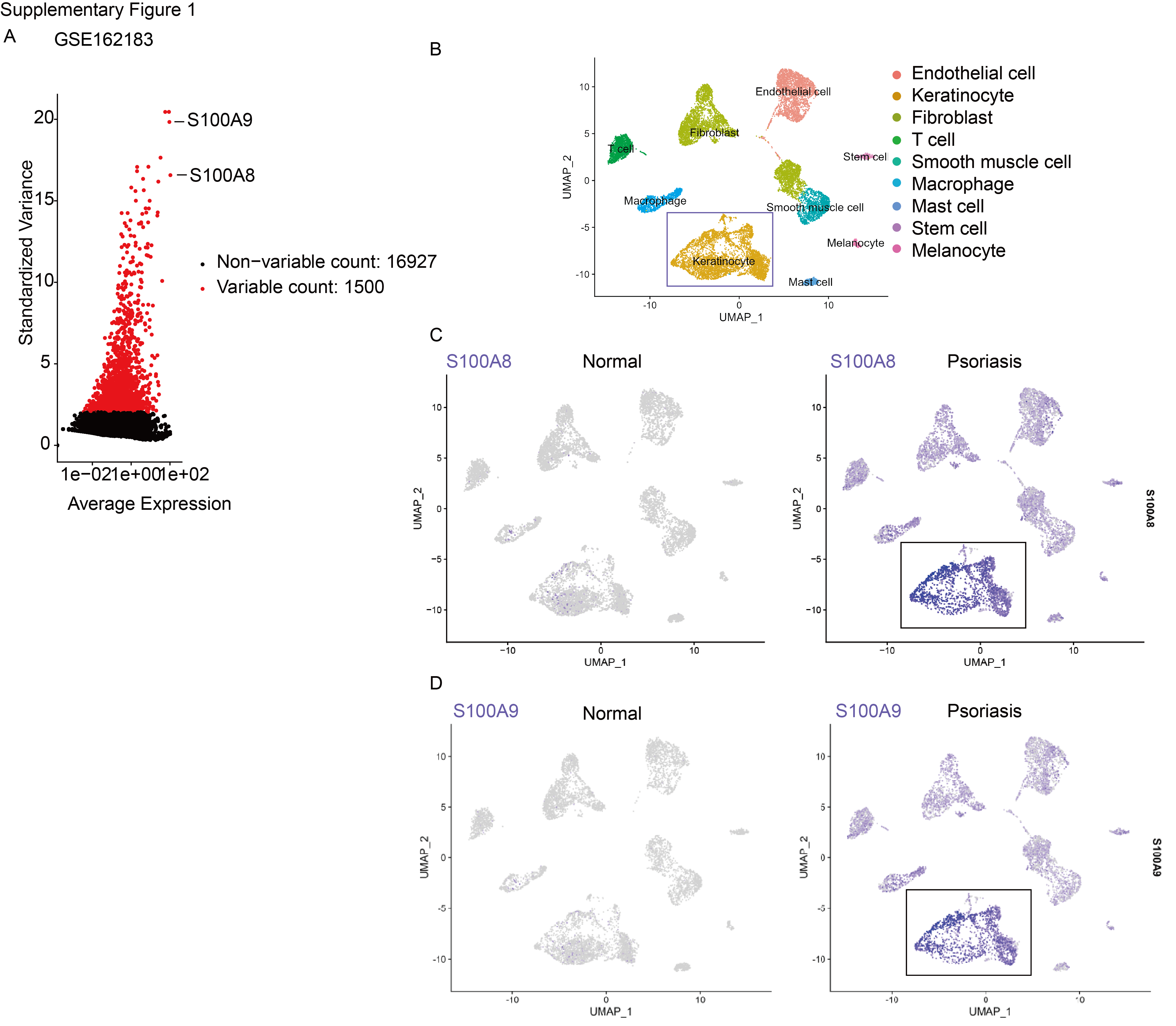

Supplement: Supplementary file 1 — Supplemental Figure 1 [file 41419_2024_6545_MOESM1_ESM.png]

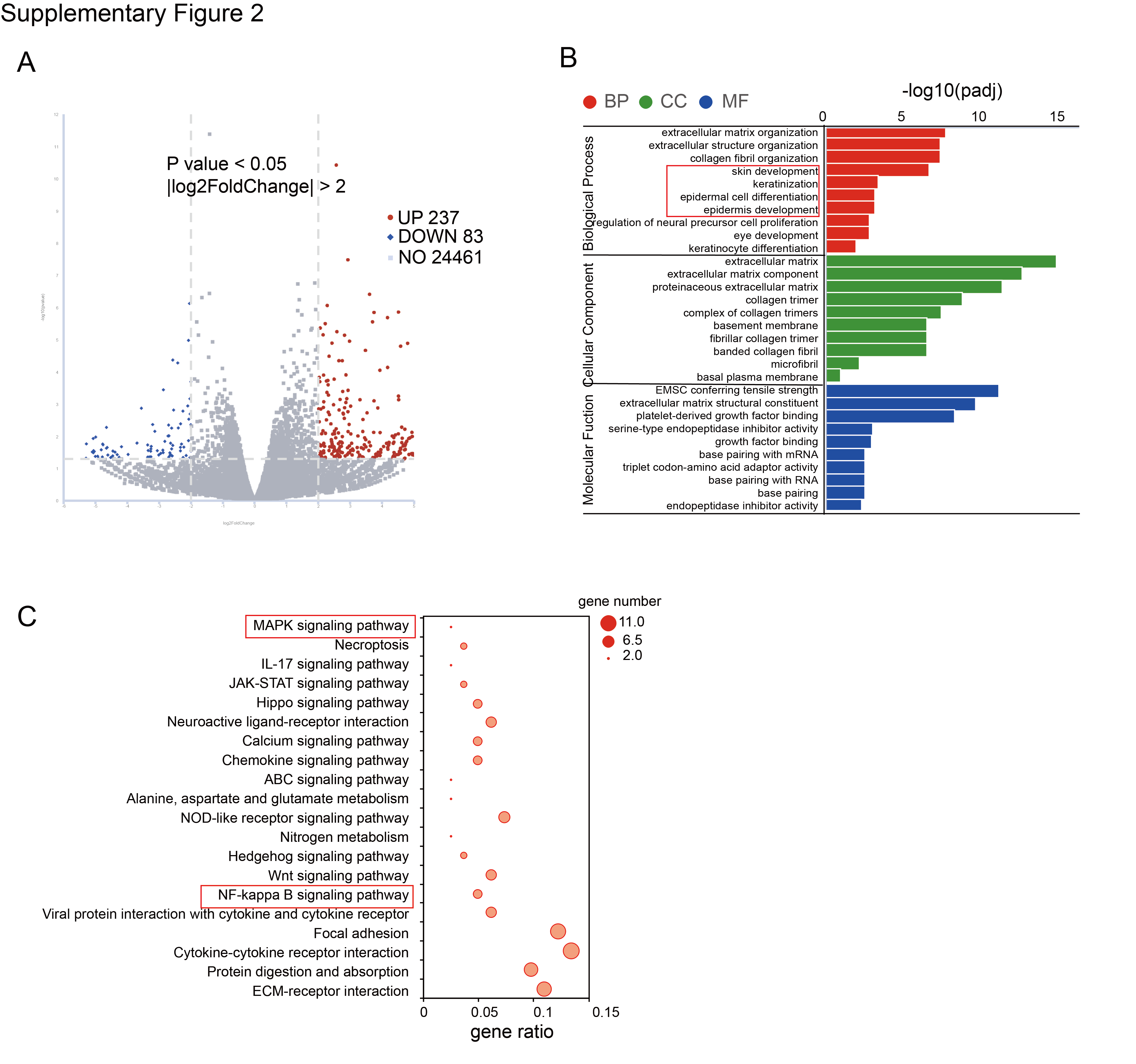

Supplement: Supplementary file 2 — Supplemental Figure 2 [file 41419_2024_6545_MOESM2_ESM.png]

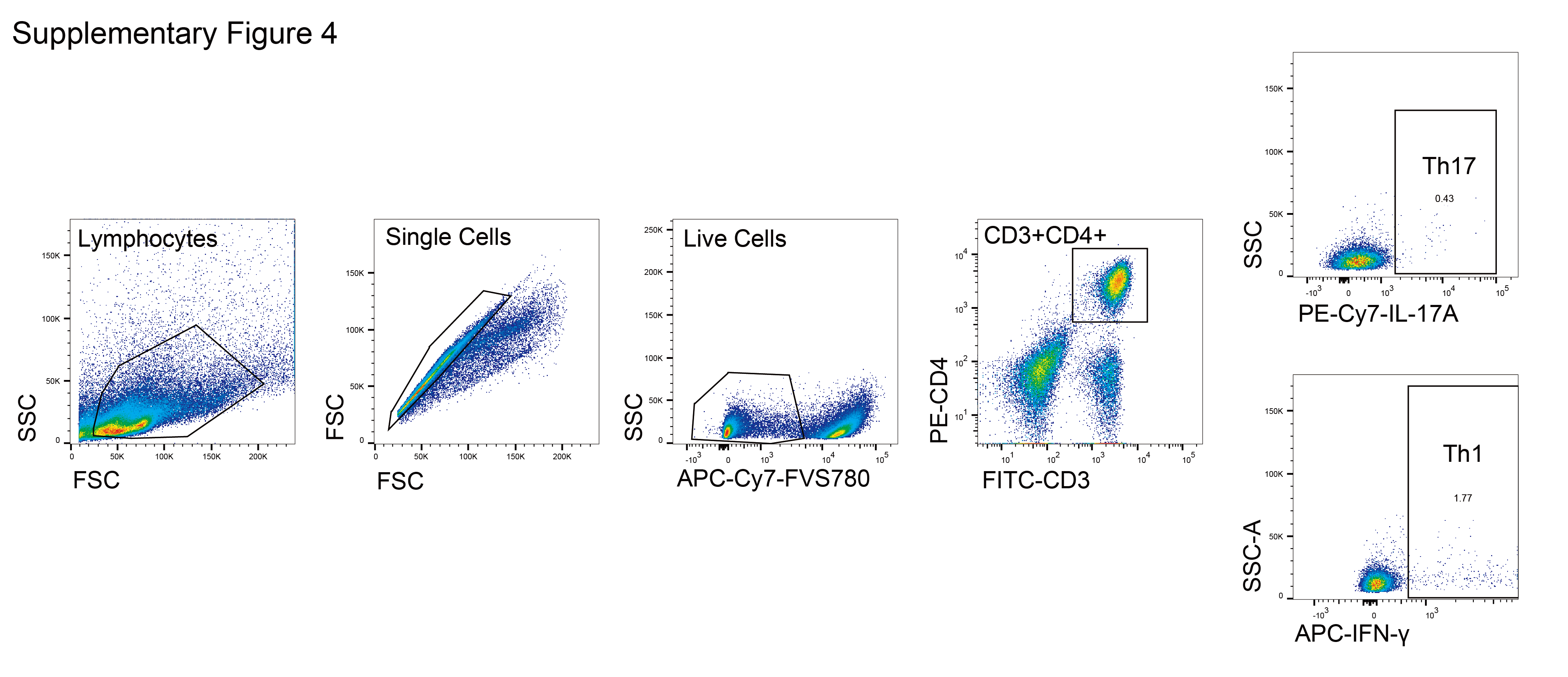

Supplement: Supplementary file 4 — Supplemental Figure 4 [file 41419_2024_6545_MOESM4_ESM.png]
