# Supplementary material for: Gasdermin E promotes translocation of p65 and c-jun into nucleus in keratinocytes for progression of psoriatic skin inflammation
Source: Cell Death Dis. 2024 Mar 1;15(3):180. doi: 10.1038/s41419-024-06545-5 (PMC10907691; doi:10.1038/s41419-024-06545-5)
Supplement: Supplementary file 5 — Supplemental Figure Legends [file 41419_2024_6545_MOESM5_ESM.docx]

Supplemental Figure Legends

Supplementary Figure 1 S100A8 and S100A9 expression and distribution in psoriasis patients.

All results presented were based on a re-analysis of GSE162183 dataset using R studio software. (A) S100A8 and S100A9 ranked among the top 10 highly variable genes in psoriasis. (B) Presentation of cell clusters from 3 normal skin from healthy people and 3 lesional skin from psoriasis patients. (C) Display of the expression levels of S100A8 and S100A9 in different cell clusters. S100A8 and S100A9 are highly expressed in keratinocytes.

Supplementary Figure 2 NF-κB and MAPK pathways were enriched in the analysis between cKO mice and littermate controls stimulated with IMQ.

All presented results were based on our RNA-seq data from the epidermis of 3 Krt14^Cre/+^-*Gsdme*^fl/fl^ mice and 3 Krt14^+/+^-*Gsdme*^fl/fl^ mice, both groups stimulated with IMQ. (A) The volcano plot displayed the differentially expressed genes between the two groups. *p* < 0.05, |log2FC| > 2. (B) GO enrichment analysis of the two groups revealed that differentially expressed genes are related to skin functions, including skin development, keratinization, epidermal cell differentiation, and epidermis development. (C) KEGG enrichment analysis indicated that NF-κB and MAPK pathways were activated. BP: biological process, CC: cellular component, MF: molecular function.

Supplementary Figure 3 All replications of western blotting bands.

Supplementary Figure 4 Gating strategy of lymphocytes from spleen tissue.
